# Prevalence and factors associated with prediabetes and diabetes in fishing communities in penang, Malaysia: A cross-sectional study

**DOI:** 10.1371/journal.pone.0228570

**Published:** 2020-02-10

**Authors:** Fairuz Fadzilah Rahim, Surajudeen Abiola Abdulrahman, Siti Fatimah Kader Maideen, Abdul Rashid

**Affiliations:** 1 Department of Public Health Medicine, RCSI & UCD Malaysia Campus (formerly Penang Medical College), George Town, Penang, Malaysia; 2 Health Education England, Cambridge, United Kingdom; Makerere University School of Public Health, UGANDA

## Abstract

**Background:**

Diabetes is a metabolic disorder, characterized by hyperglycemic state of the body. A silent killer, which can take the lives of victims if undiagnosed at the earliest stage. Prediabetes has become an important health concern across countries due to its huge potential for the development of diabetes and other complications. The objectives of this study were to determine the prevalence of prediabetes and diabetes and its associated factors among rural fishing communities in Penang, Malaysia.

**Methods:**

A cross-sectional study was conducted among fishing communities in Southwest District of Penang, Malaysia from August to November 2017. Blood sample (finger prick test) and physical examination were performed on sample of 168 participants consented in this study. Pre-validated Malay versions of International Physical Activity 7 (IPAQ-7) and Perceived Stress Scale (PSS) questionnaires were used to assess the level of physical activity and stress levels of the participants. Multinomial logistic regression models were fitted to identify factors associated with prediabetes and diabetes.

**Results:**

The prevalence of diabetes and prediabetes were 19.6% (95% CI: 14.3, 26.4) and 10.12% (95% CI: 6.4, 15.7) respectively. The median physical activity (interquartile range) in MET-minutes per week for those with diabetes (1071.0 (2120.0)) and prediabetes (1314.0 (1710.0)) was generally lower as compared to non-diabetes. Majority reported moderate stress (57.3%) from PSS system. Abdominal obesity, family history of diabetes and being hypertensive were significant factors associated with diabetes; while older age, bigger waist circumference and self-perceived poor routine diet were factors associated with prediabetes.

**Conclusions:**

The screening for prediabetes in this population gives the opportunity to implement lifestyle interventions at the earliest possible, which could prevent the development of diabetes. The identification of diabetic individuals provides an opportunity to conduct health promotion and education to ensure good metabolic control and hence reduce the risks of complications.

## Introduction

Diabetes is a metabolic disorder, a chronic disease, which is often characterized by hyperglycemic state of the body. This is often due to either deficiency in the insulin secretion, resistance to insulin action or inadequate response to the insulin secretion. Worldwide, it was estimated that about 451 million adults aged 18–99 years old were suffering from diabetes in the year 2017, approximating to about 8.4% of the global population, and it is increasing rapidly in low- and middle-income countries. Diabetes prevalence is expected to increase to 693 million by 2045, equaling to 9.9% of the same population [1]. Diabetes and impaired glucose tolerance have been significantly associated with high morbidity and mortality. In 2016, diabetes was reported to have led to 1.6 million deaths globally. Meanwhile, estimated deaths attributable to high blood glucose was 2.2 million in 2012 [2].

The latest World Health Organization (WHO) assessment for years of 2000–2016 showed diabetes as one of the major disease burden worldwide [3]. There is an alarming increase in the prevalence of diabetes in Malaysia as reported by the National Health and Morbidity Survey [4]. A study in Malaysia found the highest prevalence of Type 2 diabetes (T2D) among Indians, followed by Malays and Chinese [5]. Diabetes substantially increased the risk of cardiovascular diseases, and had accounted for 35% in total Malaysian mortality [6]. The factors associated with T2D include obesity, central obesity, hypertension, age, family history and perceived stress [7] whereas being an active smoker, poor diet and physical activity have been recognized as traditional factors for diabetes [8–10].

While prevalence, factors, management and economic implications of diabetes have been extensively studied in Malaysia, there is still scarcity of studies concerning the magnitude and trend of prediabetes, particularly among rural populations, a gap which the current study aimed to address. Prediabetes known as intermediate hyperglycemia has a similar risk to diabetes and the complications [11]. Current evidence suggest that diabetes can be prevented at early stage by resolving the progression of prediabetes to diabetes [12, 13]. Leaving the conditions undiagnosed has a costly public health implication. Identification of factors associated with both, especially prediabetes, and implementing interventions to tackle them are crucial to reduce health care cost and prevent lifelong disease.

In achieving the Sustainable Development Goals (SDGs) that lay the foundations of leaving no one behind, the study chose rural population to inculcate the planning that aligns with the global drives to reduce socioeconomic inequalities in health. The communities living in rural locations give very minimal importance and priority to routine medical screening because of their perceived good self-health [14]. It has been reported that among those rural populations, fishing communities reported higher mean BMI for female elderly and highest mean waist to hip ratio (WHR) in each age group for men [15]. Until now, little is known about the health status in the community. Thus, this study aimed to determine the prevalence of diabetes and prediabetes; and its associated factors in fishing communities. The findings of the study will benefit the community by enabling early detection and initiation of prompt treatment and consequently reduce debilitating complications.

## Materials and methods

### Study design and participants

A cross-sectional study was carried out in Penang, Malaysia from August to November 2017. Penang is located in north western Peninsular Malaysia. The Penang state consists of two parts; Penang Island and Seberang Perai located on the mainland with the two separated by the Straits of Malacca. Penang comprises of five districts; namely Southwest Penang Island, Northeast Penang Island, North Seberang Perai, Central Seberang Perai and South Seberang Perai. The study was conducted in the Southwest Penang Island as the district covers majority of the settlement of fishing communities.

Fishing communities are dispersed on the island. Three fishing communities were selected as the study site from simple random sampling of 14 fishing communities in the Southwest Penang Island. Due to the nature of the houses which are scattered and the unavailability of the list name of the residents in these communities, a convenience sampling was used to recruit the study participants. The inclusion criteria of this study were individuals aged 18 years and above, permanent residents of the community for at least 6 months and able to understand Malay or English language. Pregnant women were excluded from the study.

### Sample size

The sample size was determined using two proportions formula from the 6.2% T2D prevalence in data registry from 2009 to 2012 for the whole Penang state [16]. The prevalence of T2D was estimated to have three times increased (18.6%) for the fishing communities, consistent with current national data and considering the increased burden of the disease yearly, underreporting of the disease in the fishing communities, and majority of them being Malays [5]. It had been reported the prevalence of undiagnosed diabetes (prediabetes) was higher in rural as compared to urban areas [4]. This study estimated that a sample of 156 will provide at least study power of 90% to detect the three times difference with a significance level of 5%. Final sample size required was 172 participants after consideration of 10% non-response rate.

### Data collection

Data collection was carried out by the researchers and trained research assistants (consisting of a medical practitioner and 30 medical students for each study site). All research assistants underwent four days training on research methodology and data collection. They were given a Research Guide and Training Manual (S1 File), and had a mock-practical on appropriate ways to obtain information from participants. Prior to the study, the chiefs of the communities were approached and informed about the details of the study and their cooperation was sought. Once agreed, the chief circulated the information concerning the study to the community. A banner about the study was placed at the fence of the mosque, a place not only for religious activities but also for community gatherings; to sensitize the community about the study. Few counters were set up, which comprised of registration and taking consent, measurement of blood glucose, blood pressure and physical examinations, questionnaire and counselling. First, the prospective participants were screened for eligibility. Upon fulfilling the eligibility criteria, they were explained in detail about the study and were given participant information sheet. After understanding the information, they had the opportunity to ask questions about the study, the benefits and risks in taking part in the study. A written informed consent was obtained from all the study participants after the participants were satisfied that their participation was entirely voluntary and all their queries were answered. Thereafter, a finger prick test and physical examinations (comprising mainly anthropometric measurements) were done. The physical examinations of the participants were taken by the research assistants by following the guidelines provided in the Research Guide and Training Manual (S1 File). In order to minimize bias, examination that involved measurements (such as height, weight, waist and hip circumference, skinfold test, and PEFR) were taken by same research assistants. Self-administered questionnaire was given to each participant, whereby some elderly with reading difficulty were assisted. A copy with the individual’s measurements and readings from the health assessment was given to each participant for their record. Those with borderline reading or abnormal level were advised to seek medical advice in health clinic for further confirmation.

### Ethics approval and consent to participate

Ethical approval was obtained from the Joint Penang Independent Ethics Committee (JPEC) prior to study commencement with approval number JPEC 17–0040. For each participant, there were two copies of informed consent; one for the participant to bring back, and one copy was kept by researcher for documentation. The participants were advised of their right to voluntary withdrawal at any time without any consequences or penalties. All information was kept as confidential in a locked cabinet.

### Measurements and questionnaires

The questionnaire consisted of six sections in Malay language. The first section was on the socio-demographic characteristics, which included age, gender, marital status, education level, medical history, smoking status etc. The second part was on dietary assessment which consisted of two items on daily servings of fruits and vegetables, respectively rated on a five- scale measurement from not every day (scored 0), 1 serving per day, 2 servings per day, 3 servings per day, 4 servings per day and 5 or more servings per day (scored 5). The total score of the two items ranged from the minimum score of 0 to maximum score of 10. Section three was the short version of International physical activity questionnaire 7 (IPAQ-7) used to assess the level of physical activity of the participants for the past seven days. The questionnaire was downloaded from the IPAQ website; www.ipaq.ki.se. The website provides the questionnaire in short and long version, and also in multiple languages. The validated Malay version of IPAQ-7 consists of seven items and measures the metabolic equivalent of task (MET) in minutes. Three categories of low, moderate and high physical activity were generated from a scoring protocol prefilled with formulae [17]. Some local studies evaluated the reliability and validity of the short version which was acceptable and good [18, 19].

Section four assessed the perception of stress throughout the preceding month using a validated Malay version of Perceived Stress Scale (PSS) questionnaire [20]. The PSS consist of 10 items rated on a five options scale of never (score 0) to very often (score 4). There were four reverse items (number 4, 5, 7 and 8). The sum total scores were divided into three categories of low stress (score 0–13), moderate stress (score 14–26), and high perceived stress (27–40).

Section five consisted of two items, assessing the participant’s perceptions on risky behaviors such as poor diet, smoking, poor physical activity. The last section was on physical examination. Blood pressure was taken thrice, at three different time points, and the average blood pressure was recorded. Peak Expiratory Flow Rate (PEFR) which measures the airflow and obstruction in the airway; was measured twice, and the average reading was recorded. PEFR interpretation was done taking into consideration age, gender and height of participants. The approach for anthropometric measurements was according to practicality for survey in communities. The measurements included examination of weight (using pre-calibrated weighing scales) and height from which the Body Mass Index (BMI) was derived; hip and waist circumference. Skin folds test using caliper at several body parts was done to measure the fat under the skin, thus estimating the body fat percentage.

The measurement of blood glucose was done and recorded as Fasting Blood Sugar (FBS), if the participants fasted at least 10 hours prior to the test. The Random Blood Sugar (RBS) was done for those who did not fast prior to the data collection. MediTouch blood glucose monitor (MEDISANA^®^) was used to measure participants’ blood glucose, and results served as screening purpose and not for diagnosing diabetes. The MediTouch blood glucose test strips is plasma calibrated glucometer that is specifically designed to easily compare with laboratory test results [21]. Control tests using glucose control solution were run periodically, especially at the start of the day and on suspicion of malfunction (recommended by manufacturer) to ensure the meter and test strips are reliable and functioning well. The normal reading for FBS in an adult without diabetes ranged between 3.9 and 6.7 mmol/L. Meanwhile blood glucose reading for RBS in an adult without diabetes is expected to be less than 7.8 mmol/L. However, those participants with diabetes need to consult their personal doctor for range level that valid for them.

Participants who reported they have been previously diagnosed of T2D were defined as diabetes group. The researchers verified diabetes group based on participants self-reports on medication and treatment. Meanwhile, prediabetes was defined as participants who were newly detected to have high blood glucose from the finger prick test, and were thus classified under prediabetes group. The remaining participants were non-diabetes group with normal blood glucose, which was also defined as the reference group in the data analysis.

Hypertension group included those participants who reported they were previously clinically diagnosed with hypertension, and/or on medication and treatment. Those with uncontrolled hypertension and new cases of elevated blood pressure were advised to seek further treatment in clinics or hospitals. The blood pressure meter was calibrated by the research team (led by a medical practitioner) according to the manufacturer’s operation manual and recommendations [22].

### Variables definition

1. Smoking status: Five categories of non-smoker (never a smoker), ex-smoker (one year or more not smoking), current smoker (smoking at least one cigarette a day), occasional smoker (smoking but not every day), and smoking cessation (currently on smoking cessation).
2. Cigarettes per day: Average number of cigarettes per smoker per day.
3. Pack year smoking: Measuring smoking intensity by multiplying the packs of cigarettes per day (1 pack equal to 20 cigarettes) by the number of years the participant has smoked.
4. Body Mass Index: Measures nutritional status in an adult by dividing participant’s weight (in kilograms) with the height (in meters squared).
5. Abdominal obesity: Measuring central obesity in which the excessive abdominal fat around participant’s stomach and abdomen. Abdominal obesity is defined as waist circumference measurements of more than 90 cm for men and more than 85 cm for women.
6. Body fat percentage: Measuring body fat by dividing total mass of fat by total body mass multiplied by 100.

### Data analysis

Data were entered into excel, imported and analyzed using Stata Version 13.0 [23]. Descriptive analysis of socio-demographic characteristics and factors related with prediabetes and diabetes were done. Frequency and percentages were displayed for categorical variables. Means and standard deviations were displayed for normally distributed numerical variables, while medians and inter quartile ranges (IQR) were displayed for skewed numerical variables.

The best analysis to address the factors associated with prediabetes and diabetes in respect to normal blood glucose was multinomial logistic regression due to the nature of its outcome (three nominal categories). Univariable multinomial logistic regression was done to determine the association between the individual factors and outcome variable; diabetes group, prediabetes and non-diabetes groups. Selection of variables from univariable to multivariable model was based on those clinically meaningful variables of *p* value less than 0.25. Multivariable multinomial logistic regression was done, adjusting for age, gender, race, family history of eye disease and smoking, smoking status, number of pack year, and physical examinations, to determine the association of multiple factors to prediabetes and diabetes, when non-diabetes was the reference group. The analysis was run by backward stepwise and forward, and enter method for the chosen variables.

Overall fitness of the model was evaluated for each logit function; diabetes and prediabetes. Model is considered good and fit if (1) *p* value more than 0.05 from the Hosmer-Lemeshow test; (2) overall correctly classified percentage more than 70%; and (3) area under the receiving operating curve (ROC) more than 70% [24].

Individual model fitness was assessed to check for possible outliers or influential. The regression diagnostic test showed outliers if (1) the percent changes for the estimated logistic probability more than 20%; (2) leverage more than 0.5; (3) covariate pattern largely deviates from other cases; (4) Hosmer and Lemeshow Delta chi-squared influence statistic more than 4; (5) Hosmer and Lemeshow Delta-D influence statistic more than 4; and (6) pregibon Delta-Beta influence statistic more than 1 [25, 26].

Best model was selected based on clinically meaningful, parsimonious and good overall fitness. Final model of multivariable multinomial logistic regression was reported in relative risk ratio (RRR) and its 95% confidence interval (95% CI).

## Results

### A. Prevalence of prediabetes and diabetes

Total number of participants in the study was 168 with response rate of 97.67%. Among these, 19.6% (95% CI: 14.3, 26.4) were known diabetes, and 10.1% (95% CI: 6.4, 15.7) were newly identified as having abnormal glucose level from this study and defined as prediabetes group.

### B. General characteristics of participants

The mean age of participants in this study was 52.63 (SD: 14.19), and comparatively similar across the three groups (Table 1). The diabetes and non-diabetes group had similar pattern of demographic characteristics where female, Malay, married and those with secondary education were majority. The exception was in the prediabetes group where males were more than females. Majority of the participants were working with a mean monthly income of USD505 (RM2024.23), which included twenty-nine of the participants under not working category (36.71%) who had income from either pension or financial assistance from a Muslim endowment fund.

**Table 1:** General characteristics of participants (N = 168).

Characteristics	Total	Frequency (%)		
		Diabetes	Prediabetes	Non-diabetes
Number (%)		33 (19.64)	17 (10.12)	118 (70.24)
Age (n = 168) ^a^	52.63 (14.19)	57.91 (10.21)	55.76 (12.04)	50.75 (15.02)
Gender				
Male	92 (54.76)	15 (45.45)	10 (58.82)	67 (56.78)
Female	76 (45.24)	18 (54.55)	7 (41.18)	51 (43.22)
Race				
Malay	158 (94.05)	31 (93.94)	16 (94.12)	111 (94.07)
Chinese	6 (3.57)	1 (3.03)	1 (5.88)	4 (3.39)
Indian	3 (1.9)	1 (3.03)	0 (0)	2 (1.69)
Others	1 (0.60)	0 (0)	0 (0)	1 (0.85)
Marital status				
Married	133 (79.17)	25 (75.76)	13 (76.47)	95 (80.51)
Single	17 (10.12)	1 (3.03)	3 (17.65)	13 (11.02)
Divorced/Widowed	18 (10.71)	7 (21.21)	1 (5.88)	10 (8.47)
Highest education				
Informal	2 (1.19)	1 (3.03)	0 (0)	1 (0.85)
Primary	50 (29.76)	11 (33.33)	6 (35.29)	33 (27.97)
Secondary	78 (46.43)	18 (54.55)	9 (52.94)	51 (43.22)
Tertiary	38 (22.61)	3 (9.09)	2 (11.76)	33 (17.96)
Working status				
Working	89 (52.98)	15 (45.45)	10 (58.82)	64 (54.24)
Not working	79 (47.02)	18 (54.55)	7 (41.18)	54 (45.76)
Monthly income (n = 119) ^a^	2024.23 (1547.26)	1466.00 (1497.13)	1916.67 (1389.20)	2178.18 (1570.00)
Family history of comorbidities				
None	23 (13.69)	3 (9.09)	2 (11.76)	18 (15.25)
Diabetes	87 (51.79)	25 (75.76)	9 (52.94)	53 (44.92)
Kidney	9 (5.36)	0 (0)	0 (0)	9 (7.63)
Smoking	84 (50.00)	17 (51.52)	6 (35.29)	61 (51.69)
Eye disease	25 (14.88)	5 (15.15)	5 (29.41)	15 (12.71)
Cancer	17 (10.12)	4 (12.12)	0 (0)	13 (11.02)
Heart disease	35 (20.83)	11 (33.33)	3 (17.65)	21 (17.80)
Nerve disease	11 (6.55)	3 (9.09)	0 (0)	8 (6.78)
Pulmonary disease	7 (4.17)	1 (3.03)	0 (0)	6 (5.08)
Hypertension	90 (53.57)	21 (63.64)	6 (35.29)	63 (53.39)
Joint disease	28 (16.67)	4 (12.12)	4 (23.53)	20 (16.95)
Patients comorbidities				
None	92 (54.76)	3 (9.09)	8 (47.06)	81 (68.64)
Kidney	3 (1.78)	2 (6.06)	1 (5.88)	0 (0)
Eye disease	28 (16.77)	7 (21.21)	4 (23.53)	17 (14.53)
Cancer	1 (0.60)	0 (0)	0 (0)	1 (0.85)
Heart disease	6 (3.57)	3 (9.09)	0 (0)	3 (2.54)
Nerve disease	6 (3.57)	3 (9.09)	1 (5.88)	2 (1.69)
Pulmonary disease	1 (0.60)	0 (0)	0 (0)	1 (0.85)
Hypertension	46 (26.79)	23 (69.70)	5 (29.41)	17 (14.41)
Joint disease	27 (16.17)	4 (12.50)	4 (23.53)	19 (16.10)
Smoking status				
Non-smoker	97 (57.74)	19 (57.58)	9 (52.94)	69 (58.47)
Ex-smoker	25 (14.88)	9 (27.27)	5 (29.41)	11 (9.32)
Current smoker	31 (18.45)	3 (9.09)	2 (11.76)	26 (22.03)
Occasional smoker	13 (7.74)	1 (3.03)	0 (0)	12 (10.17)
Smoking cessation	2 (1.19)	1 (3.03)	1 (5.88)	0 (0)
Smoking activities				
Smoking duration (n = 59)^a^	22.42 (15.30)	21.82 (14.32)	21.00 (13.70)	22.83 (16.11)
Cigarettes per day (n = 60)^b^	10.00 (15.00)	20.00 (30.00)	20.00 (25.00)	20.00 (28.00)
Pack year smoking (n = 59)^a^	10.00 (17.30)	17.5 (19.8)	20.00 (13.00)	9.5 (17.4)
Anthropometric measurements				
Body Mass Index ^a^	26.93 (5.05)	28.51 (6.10)	28.68 (4.62)	26.24 (4.66)
Waist circumference (inch)^a^	36.28 (4.92)	38.95 (5.14)	38.00 (3.82)	35.28 (4.67)
Hip circumference (inch)^a^	41.37 (4.40)	42.11 (4.84)	42.76 (3.88)	40.97 (4.31)
Abdominal obesity	100 (59.52)	31 (93.94)	12 (70.59)	57 (48.31)
Body fat percentage ^a^	29.06 (8.53)	32.82 (5.60)	30.16 (9.87)	27.88 (8.72)

^a^ Mean (SD);

^b^ Median (IQR)

Less than 15% of the participants had no family history of comorbidities. Most of the participants had hypertension, eye disease and joint pains. More than 40% were either ex-smokers or current smokers and two of the participants were on tobacco cessation therapy.

### C. Assessment on physical activities, stress and diet

The median physical activity level of participants was 1540.00 (IQR: 2984.00) MET-minutes per week (Table 2). Those in diabetes group reported lowest physical activities 1071.00 (IQR: 2120.00) MET-minutes per week followed by prediabetes and non-diabetes groups. In terms of IPAQ category, most of the participants were in the high physical activity category (38.10%), however 39.39% in the diabetes group had low physical activity and 35.29% each for prediabetes had low and moderate physical activity levels.

**Table 2:** Assessment of possible factors associated with prediabetes and diabetes (N = 168).

Characteristics	Total	Frequency (Percentage)		
		Diabetes	Prediabetes	Non-diabetes
Number (%)		33 (19.64)	17 (10.12)	118 (70.24)
IPAQ ^a^	1540.00 (2984.00)	1071.00	1314.00 (1710.00)	1675.50 (2987.00)
Low	48 (28.57)	(2120.00)	6 (35.29)	29 (24.58)
Moderate	56 (33.33)	13 (39.39)	6 (35.29)	40 (33.90)
High	64 (38.10)	10 (30.30) 10 (30.30)	5 (29.41)	49 (41.53)
PSS	13.83 (5.37)	12.85 (5.33)	13.07 (6.70)	14.21 (5.20)
Low	70 (42.68)	19 (57.58)	7 (46.67)	44 (37.93)
Moderate	94 (57.32)	14 (42.42)	8 (53.53)	72 (62.07)
Diet ^b^	4.47 (1.82)	4.39 (1.84)	4.76 (1.95)	4.45 (1.81)
Self-perceived risky behaviors				
Poor physical activity	75 (45.18)	19 (57.58)	5 (31.25)	51(43.59)
Poor diet	101 (60.84)	25 (75.76)	13 (81.25)	63 (53.85)

^a^ Median (IQR) of MET-minutes per week;

^b^ Mean (SD)

The means PSS fall on low stress levels for diabetes and prediabetes groups, and moderate stress level for non-diabetes group. Diet scores were about similar for all three groups, although a comparatively higher prevalence (81.25%) of poor diet was reported by prediabetes group. Most of the participants were ready to change their risky behaviors especially concerning diet (60.84%). Among 71 smokers in the sample, fewer participants from diabetes (21.03%) and prediabetes (37.50%) groups were ready to quit smoking.

### D. Univariable multinomial regression analysis of the associated factors

Univariable (Simple) multinomial regression analysis of factors associated with T2D among diabetes group revealed a significant association with age, family history of diabetes, diagnosed with hypertension, ex-smoker, BMI, waist circumference, abdominal obesity, body fat percentage and readiness to change their diet pattern (Table 3). The significant finding for prediabetes group was waist circumference and readiness to change their diet pattern.

**Table 3:** Univariable analysis of factors associated with prediabetes and diabetes status (N = 168).

Characteristics	RRR (95% Confidence Interval) ^a^			
	Diabetes	P value	Prediabetes	P value
Number (%)	33 (19.64)		17 (10.12)	
Age (n = 168)	**1.04 (1.01, 1.07)**	**0.014**	1.03 (0.99, 1.07)	0.181
Gender				
Male	1		1	
Female	1.57 (0.73, 3.43)	0.250	0.92 (0.33, 2.58)	0.874
Race				
Non-malay	1		1	
Malay	0.98 (0.19, 4.94)	0.978	1.01 (0.12, 8.75)	0.994
Marital status				
Single	1		1	
Married	3.42 (0.43, 27.42)	0.247	0.59 (0.15, 2.36)	0.459
Divorced/Widowed	9.10 (0.96, 86.48)	0.055	0.43 (0.04, 4.82)	0.496
Highest education				
Informal/Primary	1		1	
Secondary	1.00 (0.43, 2.34)	>0.950	1.00 (0.33, 3.07)	>0.950
Tertiary	0.26 (0.07, 1.00)	0.210	0.34 (0.06, 1.83)	0.210
Working status				
Working	1		1	
Not working	1.42 (0.66, 3.09)	0.373	0.83 (0.30, 2.33)	0.723
Monthly income (n = 119)	0.9996 (0.9993, 1.0000)	0.071	0.9999 (0.9995, 1.0003)	0.554
Family history of comorbidities				
Diabetes	**3.83 (1.60, 9.19)**	**0.003**	1.38 (0.50, 3.82)	0.536
Kidney	*	0.989	*	0.992
Smoking	0.99 (0.46, 2.15)	0.985	0.51 (0.18, 1.47)	0.212
Eye disease	1.23 (0.41, 3.67)	0.715	2.86 (0.88, 9.27)	0.080
Cancer	1.11 (0.34, 3.67)	0.860	*	0.984
Heart disease	2.31 (0.97, 5.48)	0.058	0.99 (0.26, 3.75)	0.988
Nerve disease	1.38 (0.34, 5.50)	0.652	*	0.988
Pulmonary disease	0.58 (0.07, 5.02)	0.624	*	0.994
Hypertension	1.53 (0.69, 3.39)	0.297	0.48 (0.17, 1.37)	0.169
Joint disease	0.68 (0.21, 2.14)	0.505	1.51 (0.45, 5.10)	0.509
Patients comorbidities				
Kidney	*	0.999	*	0.999
Eye disease	1.58 (0.59, 4.22)	0.358	1.81 (0.53, 6.21)	0.346
Cancer	*	0.990	*	0.993
Heart disease	3.83 (0.74, 19.94)	0.111	*	0.988
Nerve disease	5.80 (0.93, 36.29)	0.060	3.63 (0.31, 42.29)	0.304
Pulmonary disease	*	0.990	*	0.993
Hypertension	**13.66 (5.54, 33.71)**	**<0.001**	2.48 (0.77, 7.92)	0.127
Joint disease	0.74 (0.23, 2.37)	0.617	1.60 (0.47, 5.45)	0.450
Smoking status				
Non-smoker	1		1	
Ex-smoker	**2.97 (1.07, 8.21)**	**0.036**	3.48 (0.98, 12.35)	0.053
Current smoker	0.48 (0.17, 1.38)	0.173	0.61 (0.15, 2.37)	0.471
Smoking activities				
Smoking duration (n = 59)	1.00 (0.95, 1.04)	0.845	0.99 (0.94, 1.05)	0.769
Cigarettes per day (n = 60)	1.02 (0.96, 1.09)	0.500	1.04 (0.97, 1.12)	0.263
Pack year smoking (n = 59)	1.00 (0.96, 1.04)	0.969	1.00 (0.96, 1.04)	0.962
Anthropometric measurements				
Body Mass Index	**1.09 (1.01, 1.18)**	**0.022**	1.10 (0.998, 1.21)	0.056
Waist circumference (inches)	**1.17 (1.08, 1.28)**	**<0.001**	**1.13 (1.02, 1.26)**	**0.024**
Hip circumference (inches)	1.06 (0.97, 1.16)	0.180	1.09 (0.98, 1.21)	0.112
Abdominal obesity	**16.59 (3.80, 72.49)**	**<0.001**	2.57 (0.85, 7.75)	0.094
Body fat percentage	**1.08 (1.02, 1.14)**	**0.005**	1.03 (0.97, 1.10)	0.299
IPAQ				
Low	1		1	
Moderate	0.56 (0.22, 1.45)	0.230	0.73 (0.21, 2.47)	0.608
High	0.46 (0.18, 1.17)	0.102	0.49 (0.14, 1.75)	0.276
PSS	0.95 (0.87, 1.03)	0.199	0.96 (0.87, 1.06)	0.434
Diet	0.98 (0.79, 1.21)	0.877	1.10 (0.84, 1.45)	0.505
Self-perceived risky behaviors				
Poor physical activity	1.76 (0.80, 3.84)	0.158	0.59 (0.19, 1.80)	0.352
Poor Diet	**2.68 (1.12, 6.43)**	**0.027**	**3.71 (1.01, 13.72)**	**0.049**

^a^RRR: Relative risk ratio.

### E. Multivariable multinomial logistic regression analysis of the significant association factors

The multivariable (multiple) multinomial regression analysis revealed significant factors associated with diabetes were abdominal obesity, family history of diabetes and diagnosed with hypertension (Table 4). Meanwhile, the significant factors associated to with prediabetes were age, waist circumference and readiness to change the poor diet.

**Table 4:** Multivariable multinomial logistic regression of factors associated with diabetes and prediabetes (N = 168).

Characteristics	RRR (95% Confidence Interval)^a^			
	Diabetes	*P* value	Prediabetes	*P* value
Number (%)	33 (19.64)		17 (10.12)	
Age (n = 168)	1.03 (0.98, 1.08)	0.301	**1.06 (1.01, 1.13)**	**0.027**
Waist circumference (inch)	1.07 (0.95, 1.21)	0.264	**1.17 (1.01, 1.34)**	**0.031**
Abdominal obesity				
No	**1**		1	
Yes	**8.29 (1.46, 47.04)**	**0.017**	0.86 (0.23, 3.25)	0.822
Family history of diabetes				
No	**1**		1	
Yes	**5.48 (1.70, 17.64)**	**0.004**	1.61 (0.50, 5.19)	0.425
Hypertension status				
No	**1**		1	
Yes	**9.66 (2.84, 32.79)**	**<0.001**	0.83 (0.20, 3.46)	0.804
Self-perceived on poor diet				
No	1		1	
Yes	3.00 (0.91, 9.87)	0.070	4.69 (1.14, 19.19)	**0.032**

^a^RRR: Relative risk ratio.

Linearity of age and waist circumference were checked and reported to be linear.

No interaction was found.

Overall fitness reported to be Hosmer-Lemeshow test (logit function 1, p = 0.897; logit function 2: p = 0.134); overall correctly classified percentage ((logit function 1, 88.0%; logit function 2: 82.6%); area under the ROC curve (logit function 1, 75.8%; logit function 2: 79.9%).

Regression diagnostic for individual fitness was performed, and no removal of individual case.

The percent changes in regression coefficient less than 20%.

## Discussion

### Summary of main findings

The prevalence of diabetes and prediabetes in this community sample from three fishing communities was 19.6% and 10.1% respectively. Participants with diabetes and prediabetes had generally lower physical activity levels compared to non-diabetes. The proportion of those with moderate stress was higher among prediabetes and non-diabetes groups. Abdominal obesity, family history of diabetes and hypertension were significant factors associated with diabetes, while older age, bigger waist circumference and self-perceived poor diet were factors associated with prediabetes.

### Prevalence of diabetes and prediabetes

The prevalence of T2D reported in this study is consistent with the findings from earlier studies [5] and a recent review of national studies conducted by Tee and Yap in 2017 [27]. According to the International Diabetes Federation, there were over 3,492,600 cases of diabetes in Malaysia in 2017,reflecting a prevalence of 16.9% [28]. Earlier, the Malaysian Ministry of Health projected that prevalence of T2D could rise to as much as 20.1% in 2020 if the major associated factors—overweight/obesity, poor diet and inadequate physical activity are not comprehensively addressed [29].

The prevalence of prediabetes in the current study was comparatively lower. Nevertheless, it corresponds to the findings of a study done among public service workers in Nigeria which reported 11.7% prevalence of prediabetes in the population [30], although the study population, demographic and sociocultural contexts differ significantly from the current study. It is projected that without any interventions among the pre-diabetic patients, they have significantly higher risk of developing diabetes in the future.

Slightly higher prevalence of prediabetes was observed in Iran. The study which was conducted among individuals aged from 15 to 75 in an urban setting showed a prevalence of prediabetes of 18.7%, which was found to be higher in elderly people [31]. In a cross-sectional study conducted by Mustafa and colleagues in 2011 among 3,879 Malaysian adults, the overall prevalence of prediabetes was reported to be 22.1% and significantly higher among women than men [32]. Studies have shown higher prevalence of prediabetes to be associated with older age and overweight/obesity [33].

### Differences in lifestyle patterns of participants

Unsurprisingly, we found generally lower physical activity levels among diabetes and prediabetes groups compared to non-diabetes group. Similarly, studies among general adult population of Malaysia reported one in two Malaysian adults with diabetes were physically inactive [34–36]. This is quite alarming, despite established evidence supporting the importance of physical activity as a key element in prevention and management of T2D [37, 38]. An intervention study in rural community showed effectiveness of the social cognitive theory-based physical activity in reducing fasting blood glucose level, body mass index, weight, and diastolic blood pressure among prediabetes group [39]. Physical activity has also been reported to delay the progression from prediabetes to diabetes, as well as reduce the morbidity and mortality associated with T2D [40]. Comparatively higher proportion of diabetes group (58%) perceived to have poor physical activity than prediabetes and non-diabetes. Whereas this suggests that they may not be oblivious of the benefits of physical activity to their condition, and the necessity to take positive action to improve their health, it is quite instructive to note that knowledge may not always translate to practice.

This study also found higher proportion of prediabetes and non-diabetes with moderate perceived stress compared to diabetes. Whereas previous studies conducted in primary care clinics and public hospitals have reported a higher prevalence of stress symptoms among Malaysian adults with T2D [41–43], it is possible that the emotional trauma of impending or anticipated life-long battle with diabetes may be precipitating higher stress among the prediabetes than diabetes who may likely be attending follow up clinics where they are supported to better cope with their condition. This may partly explain why a comparatively higher proportion of prediabetes (81%) perceived having poor diet, a lifestyle that has been established to escalate the progression of T2D and associated complications.

### Associated factors for diabetes

The current study finding that abdominal obesity, family history of diabetes and being hypertensive were significant associated factors for diabetes has a sound basis in literature [44]. Not only do these factors independently increase the risk of diabetes, there have been suggestions that interaction between these factors further potentiate the risk of diabetes. The epidemiology of abdominal obesity among Malaysian adults closely aligns with that of T2D such that both have become inseparable in terms of rising prevalence and trends across socio-demographic groups [45, 46]. To this extent, health promotion efforts on healthy diet and physical activity using risk-based or population-based approaches are bound to yield significant synergistic effects in the control of abdominal obesity and T2D among Malaysian diabetes who are generally believed to be poorly adherent to lifestyle recommendations [47, 48].

The exact pathway through which hypertension increases the risk of T2D has been extensively studied in previous literature [49, 50]. Not only does co-morbid hypertension and T2D increase the risk of complications such as cardiovascular diseases among patients, it also significantly impacts the management of both conditions, long-term survival and the health care system. It is likely that community-based efforts such as *Komuniti Sihat Pembina Negara* (KOSPEN) currently being implemented by Ministry of Health Malaysia (MOH) to improve the screening and diagnosis of non-communicable diseases (such as hypertension and T2D) would strategically impact the early detection, prompt management and prevention of complications that negatively affects the quality of life of patients.

### Associated factors for prediabetes

Age, waist circumference and self-perceived poor diet were significant factors associated with prediabetes in our study. While there are other variables associated with prediabetes, those with hypertension, hypercholesteremia, overweight, obesity and arthritis were significantly associated with prediabetes in previous studies [51]. Similar to the current study, a study done in Palau demonstrated that age and large waist circumference were factors associated with prediabetes and diabetes [52]. Poor diet control, including diets rich in carbohydrates and sedentary lifestyles contribute to the development of T2D [5]. The transitioning from prediabetes to diabetes can be affected by various factors. In a study done among pre-diabetic Mexican-Americans who were followed up for 27 months, one third of them transitioned to diabetes. The study showed that increase in participants BMI and worsening metabolic health increased the risk of transitioning to diabetes from prediabetes [53].

The screening of prediabetes in this population gives the opportunity to implement lifestyle interventions at the earliest possible, which could hinder one from developing T2D and subsequently lead to better productivity and quality of life. Imperatively, it is essential to monitor high risk populations so that comprehensive health interventions can be implemented. Improving overall metabolic health and reducing BMI would be able to improve the prognosis of diabetes and reduce the associated complications.

### Strengths and limitations

There are numerous strengths in this study. This is a first of a kind study looking at the risk factors of prediabetes and diabetes among community in fishermen communities. Besides that, the availability and usage of a standardized training manual for training of the research assistants allowed a consistent and systematic approach of gathering the data, thus increases the reliability of the measurements. Nevertheless, there are limitations that worth noting. First, the inability to recruit the study participants using probabilistic sampling limited the generalizability of the study findings to only communities in semi-urban and rural areas. Second, the nature of cross-sectional design of the study limits any potential inference of causality and temporal relationship between the variables, and increases the potential for selection bias. Third, decision on using three dependent categories (prediabetes, diabetes, and normal blood glucose) could result in having smaller values in each of the cell. Small number of samples could affect the analysis, thus the study combined some variables into fewer categories in multivariable analysis to present meaningful results. This study did not assess alcohol intake and serum lipids although these two factors could be important factors associated with prediabetes and diabetes. This study could not collect information about alcohol intake because alcohol intake is not the norm in the country and study location, thus the risk of developing into a culturally sensitive issue. Furthermore, majority of the individuals in the approached communities were Muslim and thus increased religious sensitivity around data on alcohol. Meanwhile, we could not do serum lipids test due to limitation in budgeting. Other than that, this study could not ascertain the validity of information on medical history provided by participants, as it was enquired verbally, rather than based on medical record. The recall bias of the self-reported questionnaires and measurement error could also limit the generalizability of the findings.

## Conclusions

The study identified factors associated with diabetes were abdominal obesity, family history of diabetes, and hypertension. Meanwhile, the study determined factors associated with prediabetes were older age, bigger waist circumference and self-perceived poor diet. Understanding the factors associated with prediabetes among rural community is essential so that early effective and comprehensive lifestyle interventions can be implemented. The screening of prediabetes in this population gives the opportunity to implement lifestyle interventions at the earliest possible, which could hinder one from developing diabetes; and for the diabetic patients, interventions to achieve good metabolic control, for a better prognosis and quality of life. Health promotion activities including dissemination of information in the clinics, mass media and community events are encouraged to be regularly implemented to increase the awareness of the fishermen’s community on their risk of developing diabetes and other chronic diseases.

## Supporting information

S1 File Research guide and training manual. (DOC)
