# Supplementary material for: Prevalence and factors associated with prediabetes and diabetes in fishing communities in penang, Malaysia: A cross-sectional study
Source: PLoS One. 2020 Feb 10;15(2):e0228570. doi: 10.1371/journal.pone.0228570 (PMC7010272; doi:10.1371/journal.pone.0228570)
Supplement: S1 File — (DOC) [file pone.0228570.s001.doc]

**Section 1: Socio-demographic characteristics**

- 1. **Age**

Methods:

i. Age is determined by asking the respondent verbally.

ii. Age is calculated from the year of the birth, not month of the birth. For example, those who were born in January 1988 and December 1988 should be of the same age – 25 years old effectively from January 2013. This is applicable to respondents who are more than 1 year old.

a. Formula for age is [current year] - [year of birth] i.e. 2013-1988 = 25 years old.

iii. May ask for identity card for confirmation of the year of birth.

Question:

Sir/Madam, may I know your age please?

*Encik/puan, bolehkah saya mengetahui umur anda?*

**1.2 Gender**

Methods:

i. Ask the permission to circle on the questionnaire the gender of the participant based on the physical appearance.

ii. May ask for identity card for confirmation, if necessary

Question:

May I circle ‘male/female on this questionnaire?

*Izinkan saya tandakan ‘lelaki/perempuan pada kertas soal selidik ini.*

- 1. **Marital status:**

Methods:

i. Ask the participant for their marital status and circle accordingly.

| **Race** |
| --- |
| Single / *Bujang* |
| Married/ *Berkahwin* |
| Divorced/ *Bercerai* |
| Separated/ *Berpisah* |
| Widowed/ *Duda/ Janda* |

Question:

Sir/Madam, may I know your marital status please?

*Encik/puan, bolehkah saya tahu status perkahwinan anda?*

**1.4 Race**

Method:

i. Ask the participant for verbal confirmation

Question:

What is your race?

*Apakah bangsa encik?*

**1.5 Highest Level of Education**

Method:

i. Ask the participant for their verbal confirmation

Definition:

**Illiterate/ *Tidak tahu membaca*:** Unable to read or write.

**Informal/ *Pendidikan tidak formal*:** Outside the context of a formal school/no formal education eg. ‘sekolah pondok’.

**Primary/ *Sekolah Rendah*:** Standard 1-6

**Secondary/ *Sekolah Menengah*:** Form 1-5, O-level

**Tertiary*/ Kolej/ Universiti*:** Undergraduate, masters, PHD, A-levels, matriculation, polytechnic, diploma, STPM or equivalent.

Question:

What is the highest level of education that you have received?

*Apakah pencapaian pendidikan anda yang tertinggi?*

**1.6 Occupation**

Methods:

i. Verbal confirmation is sufficient.

ii. Participants’ current occupation are recorded irrespective of duration

iii. For participants with multiple jobs;

a. Occupation recorded is the job with the highest work hours.

b. If there are two occupations with the same work hours, the occupation with the highest income is recorded.

iv. Occupational status in detail as follows:

| **Occupation** | **Code** |
| --- | --- |
| Managers | 1 |
| Professionals | 2 |
| Technician and Associated Professionals | 3 |
| Clerical Support Workers | 4 |
| Services and Sales Worker | 5 |
| Skilled agriculture, forestry, and fishery | 6 |
| Crafts and Related Trade Worker | 7 |
| Plant and Machine-Operators and Assembler | 8 |
| Elementary Occupation | 9 |
| Armed Occupation | 10 |
| Student | 11 |
| Housewife | 12 |
| Retiree/Pensioner | 13 |
| Unemployed | 14 |
| Others - none of the above | 15 |

**Managers**: Hotel manager, Restaurant manager.

**Professionals:** Doctor, Nurse (pro), Midwife (pro), Teacher, Architect, Lawyer.

**Technician and Associated Professionals:** Technicians, Operator, medical assistant, nurse (associated pro), Supervisor in factory.

**Clerical Support Workers:** Secretaries, Clerk, Data-key in operator.

**Services and Sales Worker:** Waiter, cook, shop salesperson, cashier, shopkeeper.

**Skilled agriculture, forestry, and fishery:** Fisherman, farmer

**Crafts and Related Trade Worker:** House builder, mechanic

**Plant and Machine-Operators and Assembler:** Bus/lorry driver, Taxi driver, factory worker.

**Elementary Occupation:** Cleaner, labourer, kitchen helper, garbage collector

**Armed Occupation:** Army, police, RELA.

Question:

What are you doing for living?

*Apakah pekerjaan Encik/Puan?*

**1.7 Income**

Definition:

An amount of money or its equivalent received during a period of time in exchange for labour or services, from the sale of goods or property on regular basis (more than one month), or as profit from financial investment

Methods:

i. Verbal confirmation is sufficient.

ii. Open-ended question: Monthly income is recorded to nearest Ringgit Malaysia

iii. Total income for the past 1 month.

iv. Include loans, welfare and scholarships

v. Record welfare allocated to specific individual

vi. For welfare allocated to the household, an average is calculated for each individual in the household e.g. welfare for poor

Question:

How much was your income for last month?

*Berapakah pendapatan anda pada bulan yang lepas?*

**1.8 Medical history of any family member**

Methods:

i. Verbal approach (read all the condition/ disease)

ii. May tick each that applies

iii. May tick more than one option.

Question:

Has any family member had any of the following?

*Adakah terdapat ahli keluarga pernah/sedang menghidapi kondisi ini?*

**1.9 Own medical history**

Methods:

i. Verbal approach (read all the condition/ disease)

ii. May tick each that applies

iii. May tick more than one option.

Question:

Do you have any of the following condition?

*Adakah anda pernah/sedang menghidapi kondisi ini?*

**1.10 Smoking status**

Methods:

i. Verbal confirmation is sufficient.

Question:

Do you smoke?

*Adakah anda merokok?*

**1.11 Smoking duration**

Methods:

i. Verbal confirmation approach

ii. Record in ‘years of smoking’

iii. May ask at what age the participant start smoking

Question:

How long have you been smoking? Or, At what age do you start smoking?

*Berapa lama anda sudah merokok? Atau, Pada umur berapakah anda mula merokok?*

**1.12 Cigarettes per day**

Definition:

Cigarettes per day is defined as average number of cigarettes per smoker per day.

Method:

i. Verbal confirmation approach

Question:

What is the average amount of cigarettes per day?

*Berapakah purata rokok anda hisap dalam sehari?*

**Section 2: Diet**

Methods:

i. Verbal confirmation approach

ii. Circle only one option for each question.

**Section 3: International Physical Activity Questionnaire**

Methods:

i. Verbal confirmation approach

ii. Read the question to the participant

iii. Tick each of the questions

**Section 4: Perceive Scale Score**

Methods:

i. Verbal confirmation approach

ii. Read the question to the participant

iii. Tick each of the questions

**Section 5: Self perceived on smoking habit, poor diet and poor physical activity**

Methods:

i. Verbal confirmation approach

ii. Read the question to the participant

iii. Tick questions that are relevant to the participant

**Section 6: Health screening and physical assessments**

**6.1 Blood glucose level (mmol/L)**

Dry chemistry equipment and supplies required for blood glucose tests include:

- 1. Blood Glucose Reader (Reader)
  2. FINETOUCH Lancing Device
  3. Test TIP
  4. Reader-check TIP
  5. Lancet
  6. Cotton balls
  7. Alcohol preb swab
  8. Handiplast
  9. Gloves
  10. Container.

Methods:

**The blood glucose level was measured by using the below steps [1, 2].**

1. Set out the equipment
   1. Blood Glucose Reader (Reader)
   2. FINETOUCH Lancing Device
   3. Test TIP
   4. Reader-check TIP
   5. Lancet
2. Wear glove.
3. Decide which finger you are going to obtain the blood from, preferably index finger of the right hand.
4. Press the power button on blood glucose reader
5. Remove the protective cap of the blood glucose reader by sliding the ejector button forward.
6. Insert the test TIP all the way into the blood glucose reader, and then remove the TIP case.
7. Wait for ‘Ok’ word to appear on the display.
8. Select the desired lancing depth by turning the dial.
   1. - 1: soft or thin skin
   2. 2-3 : normal skin
   3. 4 : hard or thick skin
9. Insert the lancet into the FINETOUCH Lancing Device, and twist/remove the lancet cap.
10. Wipe the side of the finger tip that you’ve selected for pricking with an alcohol prep swab and wait until the alcohol evaporates
11. Place the lancet against the skin of the finger and press the push button.
12. Gently squeeze the finger to obtain a blood sample.
13. Confirm that ‘Ok’ word is displayed. (If Ok does not appear, push the Power button and wait until it is displayed.)
14. Gently apply the blood sample to the test TIP (Please do not push the test TIP against the skin)
15. Apply the edge of the test TIP to the blood drop until you hear a ‘beep’. Then withdraw it immediately.
16. After countdown and ‘beep’ the measurement result is displayed.
17. Use the cotton ball to blot the site where you drew the blood if it is still bleeding and apply handiplast.
18. Cover the used TIP with the TIP case and slide the ejector forward to remove it.
19. To turn off, press the POWER button for more than 1 second.
    Cover the lancet with the lancet cap and remove from the Lancing Device. Safely dispose of the used test TIP and lancet.
20. Cover the Reader with the protective cap and place it inside the carrying case.
21. Use new set of test strips fine lancet, cotton balls, alcohol preb swab, gloves with each random blood glucose taken

**6.2 Blood pressure (mmHg)**

Equipments/tools:

1. Aneroid sphygmomanometer and appropriate cuff sizes
2. Stethoscope (type 2 Litmann Classic)

**The blood pressure level was measured by using the below steps [3, 4]**

Procedure:

1. Selecting the Arm for Pulse and Blood Pressure Measurement

For the **purpose of standardization,** both pulse and blood pressure are measured in the **right arm unless specific SP conditions prohibit the use of the right arm**, or, if SPs self-report **any reason that the blood pressure procedure should not use the right arm**. If the measurements cannot be taken in the right arm, they are taken in the left arm. In all cases, if there is a problem with both arms, the blood pressure is not taken.

1. Position Sample Person for Pulse and Blood Pressure Measurements
   - 1. Ask the SP to **sit all the way to the back of the chair** so that the spine is straight.

Note: if chairs is unavailable, sit on the floor, back against the wall **with legs straight and arms supported at heart level**.

One examiner might need to hold the arm supported if there is no stool available for placing the arm.

- - 1. Instruct the SP to **rest quietly for 5 minutes** prior to blood pressure measurement.
    2. The **arm and back** should be **supported** and the **legs** should be **uncrossed with both feet flat on the floor.**
    3. The **arm** should be **bared and unrestricted by clothing** with the **palm of the hand turned upward** and the **elbow slightly flexed.**
    4. The **arm should be positioned** so that the **midpoint of the upper arm is at the level of the heart.** The location of the heart is the junction of the fourth intercostal space and the lower left sternal border.
    5. Small or short sample persons may need the chair position raised or lowered to correctly position for the arm
    6. Very tall SPs may need to place their arm on an arm rest or pillow to bring their upper arm to the correct position.

1. Locating the Pulse Points
   1. Locating the **Radial Pulse**
      1. Position SP with the right palm upward.
      2. Palpate the radial pulse on the flexor surface of the wrist, laterally, with the pads of the index and middle fingers.
   2. Locating the **Brachial Pulse** in the upper arm (for BP cuff placement)
      1. Position SP with the right palm turned upward and the arm slightly bent at the elbow.
      2. Palpate the brachial pulse in the groove between the bicep and tricep muscles above the elbow with the pads of the index and middle fingers.
   3. Locating the Brachial Pulse at the Antecubital Space:
2. The brachial pulse is traced from the bicep and tricep space until palpated in the medial aspect of the antecubital fossa. The diaphragm of the stethoscope is placed under the arrow head to listen for the BP. If the pulse cannot be felt in the arm, check the radial pulse. If no radial or brachial pulse is palpable on the right arm, use the left arm unless contraindicated. If a radial pulse is apparent, whether or not the brachial pulse can be felt, the blood pressure measurement should be attempted.
3. Blood Pressure Cuff Size and Application

To obtain an accurate BP use an accurate cuff bladder size.

The length and width of the cuff’s bladder should encircle at least 80 percent of the length of the upper arm, and 40 percent of the width of an adult’s arm.

Application of the cuff:

1. Position the rubber bladder over the brachial artery at least 1” (roughly 2cm) above the crease of the elbow.

2. Wrap the cuff in a circular manner in such a way that the wrapped cuff is smooth,

snug, and no more than 2 fingers can be fit under the cuff.

3. Check the fit of the cuff to ensure that it is secure but not tight.

1. Determine the Maximum Inflation Level (MIL)

The MIL will be obtained by palpation before any blood pressure is taken.

Determine the maximum inflation level (MIL) after the sample person has been seated and resting quietly for approximately 4 minutes. The MIL or palpatory method provides an approximation of the systolic blood pressure. The **MIL is the highest level to which the cuff should be inflated when the actual measurement is made**.

The MIL is determined as follows:

1. The aneroid manometer should be calibrated to zero before MIL is determine.
2. Locate the radial pulse in the right arm;
3. Inflate the cuff quickly to a pressure of 70 – 80 mm Hg;
4. Then inflate the cuff in increments of 10 mm Hg until the radial pulse is no longer palpable (palpated systolic);
5. Continue inflating the cuff in increments of 10 mm Hg to a final measure that is 30 mm Hg above the pressure where the pulse was last palpated (palpated systolic). This number is the MIL.
6. Note this measurement and record.
7. Rapidly deflate the cuff, confirm the return of the pulse. If unable to obtain the MIL on the first attempt, wait 1 minute and repeat the process.
8. Record in nearest 2mmHg.

Procedures for blood pressure readings using the aneroid sphygmomanometer:

Explain procedure and obtain consent

1. Select the arm for pulse and blood pressure measurement, preferably **right hand for standardization.**
2. **Position** sample person for pulse and blood pressure measurements
3. Instruct the sample person to **rest quietly for 5 minutes** prior to blood pressure measurement.
4. Prepare the sphygmomanometer.
5. Select appropriate cuff to ensure it encircles 80% the length of the upper arm, and 40% of the width of an adult’s arm.
6. **Close the valve** on the pump tubing.
7. Locate the **radial and brachial pulse** points
8. **Position the rubber bladder** over the brachial artery at least 1” (roughly 2cm) above the crease of the elbow.
9. **Wrap the cuff** in a circular manner; **ensure it is secure but not tight**.
10. Determine the maximum inflation level (MIL)
11. Place earpieces of the stethoscope into the ear canals.
12. Confirm that the stethoscope head is in the **diaphragm position**.
13. Position the diaphragm of the stethoscope over the **brachial artery pulsation just above and medial to the antecubital fossa**; and hold it firmly in place, making sure that the diaphragm makes **contact with the skin around its entire circumference.**
14. Rapidly and steadily, inflate the cuff to the MIL.
15. When the MIL is reached, open the thumb valve and smoothly deflate the cuff at a constant rate near 2-mm Hg per second (one mark per second) while listening for systolic and diastolic blood pressure sounds.
16. Watch the dial of an aneroid gauge as the pressure in the bladder falls and note the level of the manometer pressure when the first repetitive sounds are heard (Phase I) and when they disappear (Phase V).
17. Continue steady deflation at 2 mm Hg per second for at least another 10 mm Hg past where the last sounds were heard.
18. Rapidly deflate the cuff and ensure the cuff deflates completely to zero.
19. Record Phase I (the level of the pressure on the manometer at the first appearance of repetitive sounds) as the systolic blood pressure reading.
20. Record Phase V (the point at which the last sound is heard) as the diastolic blood pressure reading.
21. If Phase I or Phase V occurs between the millimeter marks on the dial, round upward to the nearest **2mmHg.**
22. **Repeat the procedure twice** with the interval between two readings at least **5 – 10 minutes** apart.
23. Calculate the average of BP readings.

Limitations:

Errors in BP measurement using a sphygmomanometer can occur for several reasons, including

1. Defective equipment, for example leaking tubing or a faulty valve.
2. Failure to ensure the dial of an aneroid gauge reads 0mmHg at rest.
3. Too rapid deflation of the cuff.
4. Use of an incorrectly sized cuff – if it is too small the BP will be overestimated and if it is too large it will be underestimated.
5. The cuff is not at the same level as the heart.
6. Poor technique, for example failing to notice when the sounds disappear.
7. Observer bias, for example expecting a young patient’s BP to be normal.

**Note: Notify researcher if any of the above occur**

**6.3 Height (cm)**

Equipments/tools:

- A portable flat board with an attached metric rule
- Steel rule
- Steel measuring tape

**Record in cm unit, 1 decimal point**

The measurement of height and weight are based on the below steps [5, 6]

**Steps to assemble measuring tool**

- Place the portable flat board on a smooth surface perpendicular to a flat surface. Check the accuracy by using the steel measuring tape.

**Steps to measure height**

1. Obtain consent
2. Instruct individual to **remove any footwear** (shoes, slippers, sandals) and **head gear** (cap, hat)
3. Allow measurement taken over veil or scarf
4. Ask person to **stand facing you to measure standing height position**
5. Ask participant to **stand with legs straight, knees together, head, buttocks and shoulder blades against the measurement surface**
6. Ask participant to **look straight ahead** and **not tilt their head upwards or downwards**
7. Read the height in centimetres at exact point with the aid of the steel rule
8. Record the measurement in the patient’s instrument

Limitations:

- Observational error
- Incorrect tool

**Note: Notify researcher if you notice any of the above occur**

**6.4 Weight (kg)**

Equipment/ tools :

- Analog weighing scale in **kg**

**Record in the nearest round number. If 0.5 rounded to the larger number.**

**Set up equipment:**

- Make sure the scales are placed on a firm, flat surface.
- Calibrate weighing scale to 0

**Do not** place the scales on:

1. carpet
2. a sloping surface
3. a rough, uneven surface

Methods:

1. Ask for consent
2. Ask the participant to **remove their footwear** (shoes, slippers, sandals, etc) and **socks.**
3. **Empty all pockets**
4. Place **weighing scale on an even and stable surface.**
5. Ask the participant to **step onto scale** with one **foot on each side of the scale**.
6. Ask the participant to:
   1. stand still
   2. face forward
   3. place arms on the side and
   4. Wait until asked to step off.
7. Make sure the observer squat directly in front of the subject to read the scale.
8. Record the weight in kilograms on the participant’s instrument.

Limitations:

- Uneven surface
- Calibration error

**Note: Notify researcher if any of the above occur**

**6.5 Visual Acuity**

Definition

Clarity or clearness of one’s vision, a measurement of how well a person can see.

Introduction:

- Visual acuity is expressed as a fraction.
- For example, when a person is able to see clearly at 20 feet what should normally be seen at that distance, his visual acuity is expressed as 20. After converting into a metric value, it is almost equivalent to a 6/6 vision in meters.
- Another example, 6/12 means a person is able to see the letter clearly at 6 meters what a normal eye should see at 12 meters.
- A 6/6 does not necessarily equal a perfect vision because a person may have poor side vision, poor eye coordination or even poor colour vision.

Equipment:

1. **Snellen chart** – Devised by a Dutch ophthalmologist Dr. Herman Snellen in 1962. Consists of a series of alphabets or numbers with the largest at the top. The letters gradually become smaller as the person being tested reads the chart downwards. If the person tested is unable to read the alphabets or numbers, an alternative chart known as the Tumbling E chart is used.
   1. It measures the ability to identify small letters with high contrast at a specific distance
   2. Limitations:
      - They do not give information about seeing larger objects and objects with poor contrast e.g steps
      - Does not inform as to whether or not meaning is obtained from visual input
      - Subject needs to be literate
2. **Tumbling E Chart** - The Tumbling E chart has the capital letter "E" facing in different directions and the person being tested must determine which direction the "E" is pointing, up, down, left, or right. Use to test visual acuity if subject is illiterate.
3. **Pinhole occluder** – This enables detection of refractive error if visual acuity is improved with pinhole occlusion.
4. **Measuring tape** – To mark 6 metres from distance of Snellen chart to person tested.
5. **Pen torch**

Methods:

The measurement of visual acuity was based on the below steps [7]

1. **Snellen chart**
2. Explain procedure and obtain consent
3. Ensure good natural light or illumination on the chart
4. Using a measuring tape, position patient 6 metres away from Snellen chart
5. Use best corrected vision to carry out test (wear spectacles)
6. Test each eye separately by asking person to close left eye with left hand palm diagonally and vice versa.
7. The smallest line he can read will be expressed as a fraction e.g 6/18 or 6/24. The upper number refers to the distance of the chart away from the patient (6 meters) and the lower number is the distance in metres at which a person with no impairment should be able to see the chart.
8. If 6/6 is not achieved, record accordingly.
9. If the patient cannot read the largest (top) letter at 6 metres, move him/her closer, one metre at a time, until the top letter can be seen – the VA will then be recorded as 5/60 or 4/60, etc.
10. If the top letter cannot be read at 1 metre (1/60), hold up your fingers at varying distances of less than 1 metre and check whether the patient can count them. This is recorded as counting fingers (CF). Record as: VA = CF
11. If the patient cannot count fingers, wave your hand and check if he/she can see this. This is recorded as hand movements (HM). Record as: VA = HM
12. If the patient cannot see hand movements, shine a flashlight toward his/her eye from four directions of a quadrant. Record this in the documentation, in the relevant quadrant, as perception of light (PL or √), or no perception of light (NPL or X).
13. Repeat the whole procedure for second eye.
14. **E Chart**

- Point to each letter on each line and ask the patient to point in the direction toward which the open end of the letter is facing
- Follow the same procedure and recording methods as per Snellen chart

**6.6 Waist circumference (cm)**

| **Gender** | **WHR** | **Label** |
| --- | --- | --- |
| Male | X ≤ 0.90 | Normal |
|  | X>0.90 | Abdominal obesity |
| Female | X ≤ 0.85 | Normal |
|  | X>0.85 | Abdominal obesity |

**Waist circumference measurement**

- Waist circumference measurement is the circumference measurement of the abdomen, mid way between the top of the iliac crest and the bottom of the rib cage.

Equipment / Tools:

1. constant tension tape (for example, Figure Finder Tape Measure)

The waist and hip circumference are measured by using the below steps [5, 6]

Procedure:

- Examiner and subject should be of the same gender.
- The examination is conducted in closed area
- This measurement should be **taken without clothing**, that is, directly over the skin.
- If this is not possible, the measurement may be **taken over light clothing**. It **must not be taken over thick or bulky clothing.** This type of clothing must be removed.
- This measurement should be **taken**:
  1. **at** the **end of a normal expiration;**

The phase of respiration determines the extent of fullness of the lungs and the position of the diaphragm at the time of measurement; it also influences the

accuracy of the waist circumference.

- 1. with the arms relaxed at the sides;
  2. at the midpoint between the lower margin of the last palpable rib and the top of the iliac crest (hip bone).

1. **Standing to the side** of the participant, **locate the last palpable rib** and the **top of the hip bone**. You may ask the participant to assist you in locating these points on their body.
2. Ask the participant to wrap the tension tape around themselves and then **position the tape at the midpoint of the last palpable rib and the top of the hip bone**, making sure to wrap the tape over the same spot on the opposite side.

*Note:* ***Check that the tape is not folded, horizontal across*** *the back and front of the participant and* ***as parallel*** *with the floor as possible.*

1. Ask the participant to:
   1. stand with their feet together with weight evenly distributed across both feet;
   2. hold the arms in a relaxed position at the sides;
   3. Breathe normally for a few breaths, then make a normal expiration.
2. Measure waist circumference and read the measurement at the level of the tape to the **nearest 0.1 cm,** making sure to keep the measuring tape snug **but not tight** enough to cause compression of the skin.
3. Record the measurement on the participant’s Instrument.

Note: Measure only once and record.

**6.7 Hip circumference**

**Hip circumference measurement:**

- Measure the circumference of the hips at the widest part of buttocks.

Equipment/Tools:

- Constant tension tape (example Figure Finder Tape Measure)

Procedure:

Ideally, this measurement should be **taken without clothing,** that is, directly over the skin. If this is not possible, the measurement may be **taken over light clothing**. It **must not be taken over thick or bulky clothing. This type of clothing must be removed.**

1. **Stand to the side** of the participant, and ask them to help wrap the tape around themselves.
2. You may ask the participant to assist you in locating these points on their body.
3. Position the measuring tape around the **maximum circumference of the buttocks**.
4. Ask the participant to:
   1. Stand with their feet together with weight evenly distributed over both feet;
   2. Hold their arms relaxed at the sides.

*Note:* ***Check that the tape is not folded, horizontal across*** *the back and front of the participant,* ***snug without constricting*** *and* ***as parallel*** *with the floor as possible.*

1. Measure hip circumference and read the measurement at the level of the tape to the **nearest 0.1 cm.**
2. Record the measurement on the participant’s instrument.

Note: Measure only once and record.

**6.8 Skinfolds test**

Skinfolds callipers for measuring percentage (%) of body fat

Definition:

The skinfold caliper measures the thickness of a fold of your skin with its subcutaneous fat. It is a clinical method used to estimate a person’s percentage of body fat, in which a pinch of skin from one of seven particular areas—biceps, triceps, subscapular, suprailiac, abdomen, thigh, and calf—is measured by a caliper.

Equipment / Tools:

1. Slim Guide skinfold caliper

Site of measurement:

- sites - triceps, biceps, subscapular and suprailiac skinfolds

Limitations:

- Parallex error
- Systematic error – calibration error
- Measurement error – incorrect technique, individual patient variation
- The ease with which a double thickness can be separated from the underlying muscle tissue varies depending on the individual.

**Record in nearest 0.5mm**

**Note: Notify researcher if you notice any of the above occur**

The skin fold test was measured by using the below steps [6, 8]

Methods:

- two examiners and subject must be of same gender
- the examination should be conducted in close area
- inform subject that the examination might be uncomfortable, but will not be painful

The protocol stipulates that each skinfold should consist of a double thickness of skin and underlying adipose (fat) tissue.

1. Ask the person to stand up, with both arms at the side and relax. For handicapped people such as those with bilateral below knee amputation, ask him to sit down with full extension of his trunk.

2. **All measurements** are taken on the **Right side** of the body. It doesn’t matter what order you do the readings in.

3. There are 4 regions chosen for measurement of skinfold thickness, which are:

a) The back of the upper arm (triceps). This is located at the **midpoint between the tip of the shoulder and elbow joint.** The fold is taken in a vertical direction directly on the centre of the back of the arm.

b) The front of the upper arm (biceps). This is taken exactly the same as the triceps, except it is taken on the **midpoint of the front of the upper arm**. The upper arm is from the tip of the shoulder to the elbow.

c) Suprailiac. **Find the anterior superior iliac spine**, **and pinch horizontally 1 cm above it.**

d) Subscapular (below the shoulder blade**). Medial to the inferior angle of scapula(shoulder blade). The skinfold is pinched at 45 degrees**.

4. If you're right handed, pinch out the skinfold with the underlying layer of fat with your left hand and hold it. Then with the calipers in your right hand, place the jaws of the calipers at the skinfold, about one cm from your left hand fingers.

5. Release the trigger of the calipers. **Do not release the fingers of the left hand while taking the readings.**

6. Measure all 4 regions and write down the readings on the calipers scale.

Limitations:

systematic error: calibration error

measurement error: incorrect technique, examiner pattern variation

**Note: Notify researcher if you notice any of the above occur**

**Body fat percentage can be calculated using the Siri formula and calculation of body density is age and sex-dependent [9].**

**Body fat % = [(4.95 / BD) - 4.50 ] x 100**

**Where, BD = C - [M x (Log10 Sum of All Four Skinfolds)] whereby C and M is constant values, according to age of person as shown below**

Male

| **Age** | **17-19** | **20-29** | **30-39** | **40-49** | **50+** |
| --- | --- | --- | --- | --- | --- |
| C | 1.1620 | 1.1631 | 1.1422 | 1.1620 | 1.1715 |
| M | 0.0630 | 0.0632 | 0.0544 | 0.0700 | 0.0779 |

Female

| **Age** | **17-19** | **20-29** | **30-39** | **40-49** | **50+** |
| --- | --- | --- | --- | --- | --- |
| C | 1.1549 | 1.1599 | 1.1423 | 1.1333 | 1.1339 |
| M | 0.0678 | 0.0717 | 0.0632 | 0.0612 | 0.0645 |

**6.9 PEFR**

Equipment/ Tools:

1. Peak Flow Meters
2. Mouthpieces
3. Measuring Chart(s)

The peak expiratory flow rate is measured by using the below steps [10]

Procedure:

In the correspondent’s house:-

***Procedures are explained beforehand, and verbal consent is taken afterwards.**

1. Write the date and time of the measurements, and correspondent’s ID at the bottom of the Measuring Chart.
2. Distribute mouthpieces to each individual*; sharing mouth pieces is* ***NOT*** *allowed*.
3. One at a time the correspondent should do the following:
   1. Sit upright and put a mouthpiece on the base of the peak flow meter.
   2. Hold the meter with the right hand by the finger grip.
   3. Slide the indicator to the bottom of the scale.
   4. Hold the meter **away** from their mouth, and **inhale** as deeply as possible.
   5. Place the mouthpiece in their mouth between their teeth, and seal it tightly with their lips.
   6. **Exhale** as quickly and completely as they can. The indicator will move up the scale; the number beside the indicator is the peak flow measurement. (When exhaling patient should make a “hah” sound, not a “tah” sound. A “hah” sound is just exhaled air; a “tah” sound is made with the tongue and will not give an accurate measurement.)
4. Have each patient repeat the test three times, then take the highest of the three readings and record it.
5. The mouthpiece is then discarded.

***Reminder for all data collection personnel and investigators:***

Those participants who screened with borderline fasting capillary whole blood glucose of 6.1 mmol/L (≥110mg/dL) or random capillary whole blood glucose 7.8 mmol/L (≥140 mg/dL); and/or blood pressure of more than 140mmHg (systolic) and/or 90mmHg (diastolic) will be referred to see a doctor.

Each participant will be given a health status card. They will be advised to bring the card to the nearest health clinic for further examinations.

**References**
